# Patients' perceptions of a rural decentralised anti-retroviral therapy management and its impact on direct out-of-pocket spending

**DOI:** 10.4314/ahs.v17i3.17

**Published:** 2017-09

**Authors:** Monique Lines, Fatima Suleman

**Affiliations:** 1 Postgraduate Student, Discipline of Pharmaceutical Sciences, School of Health Sciences, University of KwaZulu-Natal; 2 Prof Fatima Suleman, Associate Professor, Discipline of Pharmaceutical Sciences, School of Health Sciences, Westville Campus, University of KwaZulu-Natal

**Keywords:** HIV, patients perception, decentralised care, South Africa

## Abstract

**Background:**

Geographical and financial barriers hamper accessibility to HIV services for rural communities. The government has introduced the nurse initiated management of anti-retroviral therapy at primary health care level, in an effort to improve patient access and reduce patient loads on facilities further up the system.

**Objectives:**

To ascertain the perceptions and satisfaction of patients in terms of the decentralised anti-retroviral policy and the direct out-of-pocket expenses of patients accessing this care in a rural setting.

**Method:**

Using a cross-sectional study design, 117 patients from five different primary health care collection points and a hospital anti-retroviral clinic were interviewed using a standard questionnaire.

**Results:**

More clinic patients walked to their clinic to collect their medicines as compared to hospital patients (71.2% versus 14.6%). Hospital patients spent more than clinic patients on monthly transport costs (ZAR71.92 versus ZAR25.81, Anova F=12.42, p=0.0009). All clinic patients listed their respective clinic as their preferred medicine collection point despite recording lower levels of satisfaction with anti-retroviral services (89% compared to 95.5%).

**Conclusion:**

Patients seem to indicate that they preferred decentralisation of HIV care to PHC level and that this might minimise out-of-pocket spending. Further studies are required to confirm these findings.

## Introduction

With an estimated 5.51 million Human Immunodeficiency virus (HIV) infected South Africans, HIV and Acquired Immune Deficiency Syndrome (AIDS) contribute significantly to the burden of disease in the country^1^. In low- and middle-income countries, health service utilisation is often determined by affordability and accessibility^2^. Since 80% of South Africans, and especially the poorest quantile of the population, rely exclusively on public sector health services^3^, the South African government has increased efforts to integrate HIV/AIDS treatment into primary healthcare (PHC) services. Task shifting and de-centralisation of care have reduced the impact of human resource shortages and improved accessibility to Antiretroviral Therapy (ART) for many affected populations in Africa^4^.

Socio-economic status remains the main predictor of access to healthcare in South Africa^5^. Based on income, South Africa has one of the highest levels of inequality in the world, with more than two thirds of rural residents and over 55% of rural households living in poverty^6^. Rural households also spend 15.4% of an annual household income of ZAR47 847 on transport^7^. It has been shown that high out-of-pocket health expenditure associated with HIV/AIDS care has a serious impact on vulnerable individuals and is likely to severely affect the wellbeing of the associated household^2^. There is also evidence to suggest that geographic inaccessibility of centralised hospital-based ART services and excessive transportation costs may contribute to patient attrition^8^ and these barriers are exacerbated in rural populations^9^.

Studies have shown the significant protective effect that decentralisation of HIV care has on the socio-economic status of households and how this facilitates access to healthcare^10^. As such, the aim of the study was to evaluate patients' perceptions and satisfaction with a decentralised Hospital HIV Programme and the perceived effect on the out-of-pocket spending of patients accessing their treatment in a deeply rural Eastern Cape community in South Africa. The Hospital team implemented a form of decentralised ART care that offered treatment initiation and support at primary healthcare (PHC) level. The team, consisting of a doctor, a pharmacy worker and a lay counsellor, would deliver ART services at the clinics once a month. Additional medicines to treat co-morbid infections and chronic diseases were also included due to the unreliable medicine supply at the PHC clinics. In March 2008, the Hospital had 242 patients on ART (0 down-referred) and by March 2009, the number had increased to 738 patients on ART (of whom 120 were down-referred).

In September 2009, donor funding was received to support dispensing at the clinics, which allowed for pre-packing of ART and enabled the potential expansion of the hospital outreach efforts. Implementation of pre-packing commenced with existing down-referred patients. A hospital team visited the clinics on designated ARV days: a doctor would consult patients needing clinical reviews, the clinic nurse was responsible for drawing the necessary blood and a lay counsellor/clinic staff dispensed the pre-packed ART to those patients requiring a treatment refill. To reduce clinic numbers and depending on the patients' clinical status and time on ART, patients received up to three months' supply of treatment. By April 2014, 4134 patients were receiving their ART through the Hospital and 3764 (91%) of these patients were receiving their ART pre-packed at a clinic of their choice.

## Methods

A cross-sectional analytical study design was employed. The study was set in the King Sabata Dalindyebo Local Municipality (KSD) of the Eastern Cape (EC) province, which is the third most populated province in South Africa (SA). KSD has a population of 451,710 living in 105 240 households^11^. The hospital selected for this study serves a rural catchment area of approximately 130,000 people and serves as a referral centre for 11 PHC Clinics. At the time of the study, 4134 patients were receiving ART through the hospital. ART was packaged as an individual patient pack at the hospital and sent to the clinics for 91% of the enrolled patients.

## Study population

A group of hospital-based patients was compared to patients accessing ART at a PHC level. Five out of the 11 down-referral clinics were selected as study sites: Clinic A (266 ART patients - furthest clinic from the hospital along a very bad dirt road), Clinic B (180 ART patients - its geographic location makes it the most difficult to access by road and/or foot), Clinic C (864 ART patients - the largest of the down-referral clinics and more easily accessible due to the tarred road), Clinic D (389 ART patients - one of the more accessible clinics along the tarred road) and Clinic E (301 ART patients - situated closer to the hospital than most clinics, accessed either by a dirt road or tarred road with a section of dirt road). The clinics were chosen based on their various sizes and accessibility to and from the hospital.

## Sample selection

In order to be sufficiently powered, a total study sample size of 94 was computed using an online calculator (http://www.surveysystem.com/sscalc.htm). This was based on the total number of patients registered for ART at the hospital (4134), confidence level of 0.95 and a confidence interval of 10. Exclusion criteria included patients that were less than 18 years of age; had been on ART for less than a month; or were not prepared to give informed consent to participate in the study.

Fifteen patients at each down-referral study site were interviewed to make up the clinic-based group. Patients from those same geographic areas who were still accessing their ART at the hospital were identified for the hospital-based group. Attempts were made to include the same number of participants in the clinic-based and hospital-based groups from each geographical area. Purposeful sampling of patients was undertaken at both the clinics and the hospital.

## Data collection

Data collection took place between 01^st^ April, 2014 and 18^th^ April, 2014. Patient demographic data such as age, gender and race was recorded. Additional patient characteristics identified included the level of education obtained, employment status, ART collection site and, for those patients collecting their ART at the hospital, the clinic located nearest to their home.

Clinic records were reviewed to retrieve data on the patients' date of ART initiation, CD4 cell count at ART initiation and most recent CD4 cell count and viral load. These record reviews were conducted at the same time as the interview since patients collected their medical records on arrival when registering for the clinic.

Individuals' socio-economic characteristics collected through the interview included: size of the household, number of known HIV positive individuals in the household and the number of these on ART, total monthly household income and sources thereof, mode of transportation to the hospital/clinic, transportation time to the hospital/clinic and any expenses incurred on the day, specifically for transportation and meals purchased. Transportation costs that were based on patient reported out-patient visits in the 30 days preceding the interview were collected. The public transport from the road-stop closest to the patients' home to the clinic/hospital was assessed. In order to better understand the factor of the distances covered by foot, walking times were also noted.

Open-ended questions on the level of satisfaction with the service provided at the ART collection site, as well as areas of dissatisfaction and suggestions for service improvements at their respective facilities were also collected.

Interviews were conducted in one of the consulting rooms in the clinic, creating a private space to facilitate an open and confidential encounter. The structured interview tool, which was pilot tested in another site, took between ten and fifteen minutes per participant.

## Data analysis

Quantitative data was coded, entered and verified using a Microsoft Excel Workbook. Descriptive statistics including means, medians, standard deviations and inter-quartile ranges were automatically calculated and summary tables generated for demographic and clinical characteristics of the sample groups. A one-way Anova (F-test) was used to test differences in means between hospital-based and clinic-based patients while Chi-square tests were done to analyse the relationship between categorical variables.

In order to assess the economic status of each household, an additional categorical variable was created, which indicated the income per household member per month. These values were based on the March 2014 figures published by Statistics South Africa^6^ and were categorised as follows:

- Below the food poverty line of ZAR400
- Between the food poverty line and lower-bound poverty line of ZAR544
- Between the lower-bound poverty line and the upper-bound poverty line of ZAR753 or;
- Above the upper-bound poverty line

Odds ratios were adjusted using multi-variate logistic regression, and the related p-value was based on the Wald test. The level of significance was set at p=0.05 and a 95% confidence interval. All data was analysed using the SAS System. Qualitative data from the three open-ended questions were analysed in Microsoft Word for key emerging themes.

## Ethical considerations

The study received full ethics approval from the Biomedical Research Ethics Committee, University of Kwa-Zulu-Natal (BE277/13). To conduct the interviews and collect patient data at the healthcare facilities, permission was sought and granted by the Hospital HIV Programme Manager and the Hospital Manager. Written informed consent was obtained separately for the interview from each participant and anonymity of the participants was maintained during the data collection, analysis and reporting of results.

## Results

In order to ensure that a sample of 94 complete questionnaires was obtained, the authors decided to over-sample the eligible population (to take into considerations withdrawal of consent once the interview had begun). Out of 123 eligible patients who were selected, all agreed to participate in the study. Six of the questionnaires had to be rejected due to missing/incomplete data, giving a response rate of 95.1% (n=117). There were 73 clinic-based and 44 hospital-based participants (Table 1).

**Table 1:** Demographic and socio-economic data comparing hospital-based and clinic-based sample groups

	All participants	Hospital patients	Clinic patients	Test value	p-value
**Male**	31 (26.5%)	12	19	Chi-square = 0.0219	0.8825
**Female**	86 (73.5%)	32	54		
**Age** **<20 years** **20 – 29 years** **30 – 39 years** **40 – 49 years** **50– 59 years** **>60 years**	7 22 41 28 10 9	37.1 years (sd 11.24) 4 10 16 10 1 3	42.2 (sd 12.73) 3 12 25 18 9 6	Anova (F=4.81) Chi-square = 5.112	0.0303 0.4024
**Education** **None** **Primary school** **Secondary school** **Matric and higher**	25 37 45 8	3 10 26 4	22 27 19 4	Chi-square = 6.47	0.0004
**Employed** **Unemployed**	7 (5.9%) 110 (94.1%)	4 (9.1%) 40 (90.9%)	3 (4.1%) 70 (95.9%)	Chi-square = 1.211	0.2711
**Duration on ART**		37.73 months	33.23 months	Anova (F=0.97)	0.3262
**Baseline CD4**	163.09	156.30	167.33	t-test (F=0.3)	0.5875
**Most recent CD4**	464.41	453.70	470.99	t-test (F=0.15)	0.6985
**CD4 increase**	351.88	339.56	359.03	t-test (F=0.23)	0.6329
**Undetectable viral** **load**	88%	84%	91%	Chi-square = 1.1694	0.2795
**Household size:** **Total** **12 years and older** **Under 12 years**	6 4 3	6 4 3	6 4 3		
**Total monthly** **household income**	ZAR1630	ZAR1653	ZAR1617		
**Monthly income per** **capita**	ZAR358.21	ZAR301.05	ZAR392.66	Anova (F=0.8)	0.3734
**Economic status** **Below the FPL** **Between the FPL** **and LBPL** **Between the LBPL** **and UBPL** **Above the UBPL**	83 (70.9%) 18 (15.4%) 8 (6.8%) 8 (6.8%)	33 (75%) 5 (11.4%) 5 (11.4%) 1 (2.2%)	50 (68.5%) 13 (17.8%) 3 (4.1%) 7 (9.6%)	Chi-square = 0.6822	0.8774

As indicated in Table 1, of the 117 participants, 31 (26.5%) were male and 86 (73.5%) were female. There was no significant difference between hospital-based and clinic-based patients in terms of gender (Chi-square = 0.0219, p=0.8825). There was a significant difference between the hospital-based and clinic-based patients in terms of their actual age (37.1 years versus 42.2 years respectively, Anova F=4.81, p=0.0303) but not when categorised into age bands (Chi-square=5.112, p=0.4024).

There were significant differences between hospital and clinic patients (chi-square=6.47, p=0.004) in terms of education. Just over 30% of the clinic-based patients had no schooling compared to 7% of the hospital-based patients. Significantly more hospital-based patients had secondary schooling compared to the clinic-based patients, 60.5% versus 26.4% respectively.

The mean CD4 cell count at initiation of ART was slightly higher among clinic-based patients (167 cells/mm^3^ versus 156 cells/mm^3^). Although the clinic-based patients had a shorter mean duration on ART (33.23 months compared to 37.73 months for hospital-based patients), the mean CD4 increase was higher in the clinic-based group, although not significantly so (t-test F = 0.23, p = 0.6329). Only seven out of the total study sample of 117 patients had some form of employment. The average monthly household income was ZAR1653 (ZAR301.05 per capita) for hospital-based patients and ZAR1617 (ZAR392.66 per capita) for clinic-based patients, predominantly sourced from either child support or pension grants (98 households and 50 households respectively). Monthly per capita income was not significantly different between the two study groups (Anova F=0.8, p=0.3734).

75% of hospital-based patients and 68.5% of clinic-based patients were living below the food poverty line of ZAR400 per month. 93% of the study participants were classified as poor i.e. living below the upper bound poverty line of ZAR753 per month. The economic status of the hospital-based patients was not significantly different from that of the clinic-based patients (Chi-square=0.6822, p=0.8774).

Mode of transport to access ART was significantly different between hospital- and clinic-based patients (Chi-square=34.9183, p=0.0001). A higher proportion of hospital-based patients used taxis (80.5% versus 28.8%) while more clinic-based patients walked to the facility for their treatment (71.2% versus 14.6%) and indicated that taxi fare was unaffordable or that they lived close by. Clinic-based patients did, on average, walk for longer periods of time than hospital-based patients although this was not significantly different (Anova F=1.67, p=0.2026).

In terms of monthly transport costs, hospital-based patients spent on average ZAR71.92, significantly more than the ZAR25.81 spent by clinic-based patients (Anova F=12.42, p=0.0009). This was specifically as a result of the transport costs associated with the PHC Clinics C and D being significantly different to those of Hospital patients (p=0.0175 and p=0.0072 respectively).

When considering all direct out-of-pocket expenditure related to accessing ART (transportation and meals while waiting), clinic-based users had a lower mean cost per month compared with hospital-based patients and this saving was statistically different for all down-referral clinic sites included in the study (Anova F=13.27, p=<0.0001). There were higher levels of satisfaction recorded amongst the hospital-based group (95.5% compared to 89%) but despite this, 100% of the clinic-based patients listed their respective clinic as their preferred ART collection point. Despite being satisfied with the level of care at the hospital, just over a fifth of the hospital-based patients would prefer to collect their ART and receive HIV care at their nearest clinic.

Positive themes consistent in both groups included the fact that patients were receiving excellent counselling and being taught a lot about HIV and their treatment. Patients were satisfied with the amount of time they were spending at their respective facility on appointment days and hospital-based patients in particular were complimentary of the queuing system, which they felt facilitated good patient flow. Hospital-based patients felt they were always informed of where they were meant to go and what they had to do on their appointment days. Hospital-based patients were largely dissatisfied with the infrastructure of the Hospital HIV clinic, complaining that the toilets were not usable and that the building was too small and too old for all of the patients to use. Hospital-based patients commented on the more private nature of their treatment collection and consultations and they were generally more positive with regards to their experiences with healthcare workers.

## Discussion

Results from this study support decentralisation of ART to PHC level. The clinic-based patients were initiated on ART at a higher CD4 cell count (167 cells/mm^3^ compared to 156 cells/mm^3^ of hospital-based patients) and also showed a slightly better increase in CD4 cell count since initiation on ART despite being on treatment for a shorter duration than hospital-based participants (33.2 months versus 37.7 months). This supports the idea that earlier initiation of ART at higher CD4 cell counts facilitates a better immune response^12^. However, without a cohort study design, it would not be possible to make a direct correlation between variables such as ease of access, or stages of disease on improved health outcomes in these patients.

The clinic-based participants had significantly lower levels of education and 59.7% were functionally illiterate. Three fifths of the hospital-based patients had achieved some level of secondary schooling. The literature shows that rural residents and individuals with low levels of educations find it more difficult to access HIV services^13^ and are less likely to utilise these services, increasing out-of-pocket spending in the long run^2^. Having a decentralised service may increase access and utilisation of services.

Over 70% of the study sample lived below the food poverty line and 93% are poor by definition. Reportedly, 98 of the households were receiving at least one child support grant and 50 households had one elderly member collecting pension money every month. Thus free PHC and free access to ART and HIV services as part of the South African government's social welfare ‘package’, may financially protect vulnerable individuals and households from the economic impact of HIV^14^.

For rural communities, access to essential health services such as ART, are often dependent on the geographical location of healthcare facilities and availability/presence of public transport^9^. The reduction in direct out-of-pocket spending seen in the clinic-based group in this study is associated with decentralisation and the pre-packing model. By moving HIV services closer to patients' homes, accessibility and associated cost of accessing ART is significantly reduced since the distance to the facility is shorter, taxi fares are cheaper and the waiting times are reduced. The lowered costs may contribute to the economic protection of the households in this rural area^15^.

Despite the many compliments for the Hospital HIV clinic staff, patients felt the facility was understaffed and criticised the nurses' handling of administrative tasks, such as providing the paperwork required for patients to transfer to another province/facility. In the Eastern Cape where the nurse vacancy rate is 67%, task shifting of HIV management from doctors to nurses has not relieved the effects of healthcare worker shortages. Clinic-based patients were also unhappy with the number of staff employed at the facilities and there were remarkably more complaints about the attitudes and poor treatment of patients by clinic nurses. Between one and three nurses were employed at the five down-referral sites included in the study, highlighting the critical shortage of qualified staff at PHC level. Since a large number of the clinic-based patients have received some care at Hospital since initiating ART, their expectations may have been raised by the availability of doctors and more experienced nurses^8^.

Availability of treatment was praised by both hospital- and clinic-based groups and a number of participants went as far as saying that they had never been turned away without their ART. The literature shows that inconsistent and unreliable supply of ART as a result of insufficient qualified staff capable of managing stock has been known to cause treatment interruptions^13^.

## Limitations

Due to time constraints, the study had a small sample size. Additionally, only five out of eleven of the down-referral clinics were included in the study. Those facilities that were not included could possibly have impacted the study results. The way in which participants and the study sites were selected may also potentially contribute to selection bias. Participant self-reporting may be subject to recall and social desirability bias. Administration of the questionnaire by interviewers who were independent of hospital staff may have reduced the risk of social desirability.

## Conclusion

Patients seem to indicate that they preferred decentralisation of HIV care to PHC level and that this might minimise out-of-pocket spending. South Africa has the largest ART programme in the world yet has only managed to provide ART to less than 40% of HIV positive individuals who are eligible for treatment^16^. Since affordability, more so than the need for healthcare services determines out-of-pocket spending^2^, a larger and more in depth analysis focusing on the economic impact of HIV on rural, impoverished communities should be done in resource limited settings presenting unique challenges to patients and the health system alike.
